# Safety and efficacy of side-to-end anastomosis versus colonic J-pouch anastomosis in sphincter-preserving resections: an updated meta-analysis of randomized controlled trials

**DOI:** 10.1186/s12957-021-02243-0

**Published:** 2021-04-21

**Authors:** Sen Hou, Quan Wang, Shidong Zhao, Fan Liu, Peng Guo, Yingjiang Ye

**Affiliations:** 1 Department of Gastrointestinal Surgery, Peking University People’s Hospital, Beijing, 100044 People’s Republic of China; 2 Laboratory of Surgical Oncology, Peking University People’s Hospital, Beijing, 100044 People’s Republic of China; 3 Beijing Key Laboratory of Colorectal Cancer Diagnosis and Treatment Research, Peking University People’s Hospital, Beijing, 100044 People’s Republic of China

**Keywords:** Side-to-end, J-pouch, Anastomosis, Sphincter-preserving resection, Rectal cancer, Low anterior resection syndrome

## Abstract

**Background:**

The application of side-to-end anastomosis (SEA) in sphincter-preserving resection (SPR) is controversial. We performed a meta-analysis to compare the safety and efficacy of SEA with colonic J-pouch (CJP) anastomosis, which had been proven effective in improving postoperative bowel function.

**Methods:**

The protocol was registered in PROSPERO under number CRD42020206764. PubMed, Embase, Web of Science, and the Cochrane Register of Controlled Trials databases were searched. The inclusion criteria were randomized controlled trials (RCTs) that evaluated the safety or efficacy of SEA in comparison with CJP anastomosis. The outcomes included the pooled risk ratio (RR) for dichotomous variables and weighted mean differences (WMDs) for continuous variables. All outcomes were calculated with 95% confidence intervals (CI) by STATA software (Stata 14, Stata Corporation, TX, USA).

**Results:**

A total of 864 patients from 10 RCTs were included in the meta-analysis. Patients undergoing SEA had a higher defecation frequency at 12 months after SPR (WMD = 0.20; 95% CI, 0.14–0.26; *P* < 0.01) than those undergoing CJP anastomosis with low heterogeneity (*I*^2^ = 0%, *P* = 0.54) and a lower incidence of incomplete defecation at 3 months after surgery (RR = 0.28; 95% CI, 0.09–0.86; *P* = 0.03). A shorter operating time (WMD = − 17.65; 95% CI, − 23.28 to − 12.02; *P* < 0.01) was also observed in the SEA group without significant heterogeneity (*I*^2^ = 0%, *P* = 0.54). A higher anorectal resting pressure (WMD = 6.25; 95% CI, 0.17–12.32; *P* = 0.04) was found in the SEA group but the heterogeneity was high (*I*^2^ = 84.5%, *P* = 0.84). No significant differences were observed between the groups in terms of efficacy outcomes including defecation frequency, the incidence of urgency, incomplete defecation, the use of pads, enema, medications, anorectal squeeze pressure and maximum rectal volume, or safety outcomes including operating time, blood loss, the use of protective stoma, postoperative complications, clinical outcomes, and oncological outcomes.

**Conclusions:**

The present evidence suggests that SEA is an effective anastomotic strategy to achieve similar postoperative bowel function without increasing the risk of complications compared with CJP anastomosis. The advantages of SEA include a shorter operating time, a lower incidence of incomplete defecation at 3 months after surgery, and better sphincter function. However, close attention should be paid to the long-term defecation frequency after SPR.

**Supplementary Information:**

The online version contains supplementary material available at 10.1186/s12957-021-02243-0.

## Background

With the progress of surgical techniques and multimodal treatment, an increasing number of patients agree to undergo sphincter-preserving resections (SPRs) [1]. SPR maintains bowel continuity, and the procedure avoids permanent stoma [2]. Some studies have indicated that SPR patients experienced a better quality of life and overall survival comparable to those undergoing abdominoperineal resections (APRs) [3, 4]. However, 80–90% of SPR patients have different degrees of anorectal disorders [5]. Low anterior resection syndrome (LARS) includes disordered bowel function after rectal resection, leading to a detriment in quality of life, and it incorporates a vast array of anorectal disorders after sphincter-preserving surgery such as fecal incontinence, urgency, clustering, and evacuation problems [6]. LARS has greatly weakened the advantages of SPR, and patients prefer APR over SPR owing to severe LARS [7].

To overcome the adverse functional outcomes of traditional straight colorectal anastomosis (SCA), some modified rectal reconstructions have been proposed. Colonic J-pouch (CJP) anastomosis has been extensively studied since it was initially proposed in 1986 [8, 9]. In this procedure, the distal colon is closed and folded, and the bottom of the J-pouch is anastomosed with the residual rectum or anal canal. CJP anastomosis is considered an optimal method for rectal reconstruction with acceptable complications [10, 11]. More importantly, CJP anastomosis increases the volume of the reservoir and effectively improves the bowel function, but some evacuation problems may persist [12–14]. Nevertheless, CJP anastomosis cannot be utilized with a narrow pelvis, bulky anal sphincters, or insufficient colon length [15]. Side-to-end anastomosis (SEA) [16] has also been used to form even smaller reservoirs; therefore, SEA is theoretically supposed to combine the advantages of the CJP anastomosis, with a wider range of applications, lesser surgical complexity, and better evacuation [17].

Looking at the last five systematic reviews or meta-analyses of SEA and CJP anastomosis studies [18–22], Hüttner et al. [21] barely investigated surgical and oncological outcomes and they failed to analyze the source of high heterogeneity even though they analyzed six publications. Four other studies [18–20, 22] contained only four or fewer publications. In addition, none of the five studies was registered with the International Prospective Register of Systematic Review (PROSPERO) or Cochrane. In the past 5 years, several RCTs have been published, and we performed an updated meta-analysis to evaluate the safety and efficacy of SEA compared with CJP anastomosis. Additional anastomotic techniques, such as transverse coloplasty, have also been developed, but safety reasons and debated functional outcomes have prevented their widespread adoption. As a result, these methods of rectal reconstructions were not included in this meta-analysis.

This study was performed according to the Cochrane Collaboration methodology and Meta-Analyses (PRISMA) [23, 24] statement.

## Methods

### Protocol and registration

In accordance with established PRISMA (Preferred Reporting of Systematic Reviews and Meta-Analyses) guidelines, the prospective protocol for this systematic review was registered in PROSPERO (registration no. CRD42020206764), available from https://www.crd.york.ac.uk/prospero/display_record.php?RecordID=206764 (Additional file 1).

### Data sources and searches

We used medical subject headings (MeSH) and free-text words to search PubMed, Embase, Web of Science, and the Cochrane Register of Controlled Trials before June 21, 2020. The search strategy was developed with a professional trial search coordinator. The search items were as follows: rectal cancer, rectal neoplasms, rectal tumors, cancer of rectum, side-to-end, end-to-side, and Baker anastomosis. We also reviewed references cited in relevant articles and several conference abstracts, including those of the International Congress of the European Association for Endoscopic Surgery and the Scientific Session of the Society of American Gastrointestinal and Endoscopic Surgeons. Details of the literature search are shown in Additional file 2.

### Selection and exclusion criteria

Studies were selected on the basis of the Patient problem, Intervention, Comparison, and Outcome (PICO) criteria as follows:

1. Population: patients with rectal cancer and treated by SPR
2. Intervention: SEA
3. Comparator: CJP anastomosis
4. Efficiency outcome measures were as follows: (a) defecation frequency (times/day), (b) the number of patients with urgency, (c) the number of patients with incomplete defecation, (d) the number of patients using pads, and (e) the number of patients using enema and medication. In addition, anorectal manometry data were also extracted to evaluate anorectal function. The safety outcome measures were as follows: (a) the number of patients with perioperative complications, (b) the number of patients with reoperations (except stoma reversal), (c) the number of patients treated with protective stoma, (d) the number of patients with relapse (< 2 year), (e) mortality (in-hospital or 30 days after surgery), (f) postoperative hospital stay (days), (g) operating time (min), and (h) blood loss (ml).

The exclusion criteria were as follows: (1) case reports, conference abstracts, and animal experiments; (2) reviews or meta-analyses; (3) case-control studies; and (4) original studies lacking available data.

### Data extraction and quality assessment

Two trained reviewers independently extracted the following data: study demographics and characteristics, including (1) first author, (2) publication year, (3) country, (4) multi-center status, (5) study duration, (6) the number of patients, (7) tumor level, and (8) tumor stage besides safety and efficacy outcomes. We used the estimated values based on the Cochrane Handbook when we could not obtain mean values and standard deviations (SDs) from the eligible studies [25, 26]. Disagreements were resolved to reach a consensus through discussion or consulting with a third investigator.

The methodological quality of RCTs was evaluated by the Jaded Scale for randomization procedures, the proportionality of the randomization method, blinding, the procedure of blinding, and statement and cause of withdrawals [27].

### Statistical analysis

We estimated outcomes by calculating the pooled RR for dichotomous variables and WMD for continuous variables by STATA software (Stata 14, Stata Corporation, TX, USA). Fixed-effects or random-effects models were applied to compute the pooled effect size with 95% CI. A *P* < 0.05 was considered statistically significant. Heterogeneity across studies was evaluated using the *I*-squared statistics (*I*^2^), and *I*^2^ > 50% was considered high [28]. Sensitivity analyses, cumulative analyses, and subgroup analyses were conducted to investigate the influence on the overall results and discover the source of heterogeneity. Moreover, funnel plots, Harbord’s test and Egger’s test were performed to assess the publication bias of the included studies [29, 30].

## Results

### Included studies

**Fig. 1:**
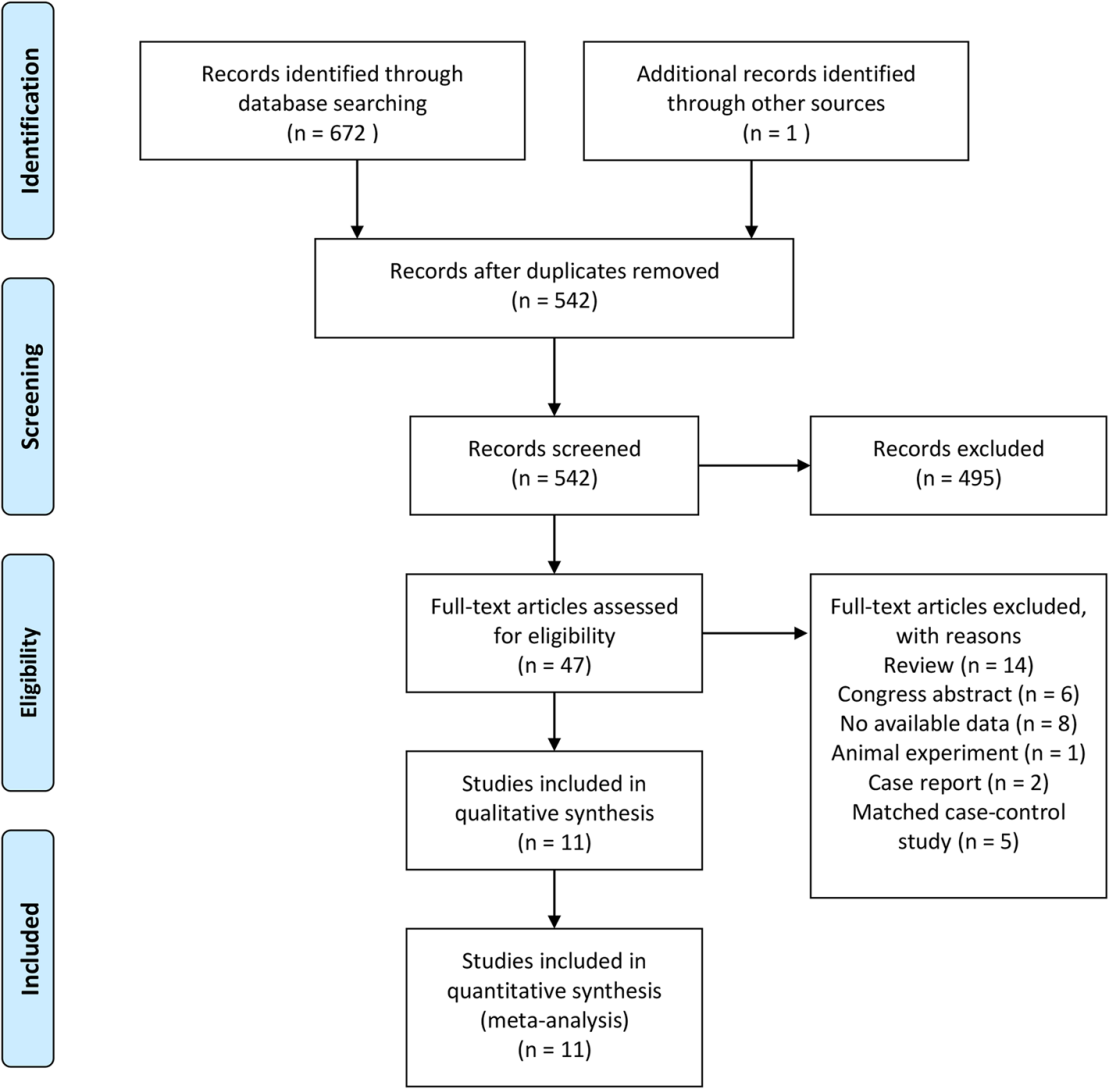
Flowchart of identification of eligible trials to include in the meta-analysis. WMD, weighted mean differences; CI, confidence intervals

The results of the literature search identified 672 studies, and 10 RCTs [31–41] (11 publications with 2 using the same cohort) were eligible for inclusion in the meta-analysis (Fig. 1).

Most of the studies were performed in Europe and Asia. The interventions of six studies [31–34, 36–38] were comparisons between SEA and CJP anastomosis, and the other four studies [35, 39–41] were based on three-arm trials. A total of 864 patients were available for this meta-analysis, and the characteristics of the included studies are summarized in Table 1. Surgical details of the included studies are summarized in Table 2.

**Table 1:** Demographic of the included trials

Study	Country	Multi-center	Duration	Group	Number	Age (year)	Sex (male/female)	Tumor level (cm)	Tumor stage	Preoperative radiotherapy	Jaded score
Marti et al. [40]	Switzerland	No	1999 to 2004	CJP	63	68.6 (30.9, 85.5)	38/25	6 (1, 11)	NR	4	6
				SEA	95	67.2 (32.3, 88.9)	62/33	7 (1, 15)	NR	3	
Parc et al. [41]	International	Yes	2007 to 2009	CJP	80	60.2 (9.7)	59/21	3 (0, 4)	NR	42	5
				SEA	87	59.6 (10.6)	52/35	2 (0, 4)	NR	50	
Rasulov et al. [39]	Russia	No	Oct. 2015 to Nov. 2017	CJP	22	57 (30, 68)	6/16	7 (5–10)	NR	11	4
				SEA	30	60 (28, 71)	18/12	7 (5–10)	NR	14	
Okkabaz et al. [38]	Turkey	No	Jun. 2009 to NR	CJP	29	58.9 (13.7)	18/11	7.9 (3.8)	NR	NR	6
				SEA	28	59.1 (11.9)	19/9	6.2 (3.8)	NR	NR	
Marković et al. [37]	Serbia	No	Jan. 2000 to Dec. 2004	CJP	40	55.25 (34, 78)	17/23	(2, 4)	2/19/19/0	0	4
				SEA	40	56.4 (35, 79)	10/30	(2, 4)	0/23/17/0	0	
Doeksen et al. [36]	Netherlands	Yes	Apr. 2002 to Jan. 2007	CJP	55	66 (33, 82)	36/19	NR	22/14/19/0	55	6
				SEA	52	66 (44, 79)	37/15	NR	12/13/15/2	52	
Akira et al. [35]	Japan	No	1999 to 2004	CJP	19	60 (45, 79)	11/8	NR	11/5/3/0	0	3
				SEA	17	62 (29, 89)	11/6	NR	6/5/6/0	0	
Jiang et al. [34]	Taiwan, China	No	Jan. 1998 to Dec. 1999	CJP	24	62.3 (3.3)	12/12	7.9 (1.5)	3/10/10/1	12	5
				SEA	24	64.9 (2.8)	15/9	8.6 (0.3)	7/8/6/3	10	
Machado et al. [33]	Sweden	No	Oct. 1995 to Apr. 1999	CJP	36	65 (38, 83)	18/18	10 (3–15)	9/13/12/0	29	4
				SEA	35	66 (50, 87)	21/14	10 (4–15)	9/15/10/0	28	
Machado et al. [32]	Sweden	No	Oct. 1995 to Apr. 1999	CJP	50	67 (38, 83)	27/23	10 (3–15)	10/17/21/0	39	4
				SEA	50	66.5 (40–87)	32/18	10 (4–15)	14/19/16/0	39	
Huber et al. [31]	Germany	No	Oct. 1995 to Oct. 1996	CJP	29	62.3	13/16	5.2 (2.5, 9)	NR	NR	4
				SEA	30	61.9	12/18	5.8 (3, 9)	NR	NR	

Continuous variables are recorded as mean (SD) or median (range); tumor stage is recorded as Dukes A/B/C/D. *CJP* colonic J-pouch, *SEA* side to end anastomosis, *NR* not reported

**Table 2:** Surgical details of included trials

Study	Group	Number	Operation time (min)	Blood loss (ml)	Protective stoma	Reversal time (m)	Anastomosis level	Reservoir size (cm)	Stapled/hand sewn	Anastomotic colon (descending/sigmoid)
Marti et al. [40]	CJP	63	NR	NR	57	5.3 (3.9, 6.1)	NR	5 (0)	NR	NR
	SEA	95	NR	NR	89	4.6 (3.5, 5.9)	NR	4 (0)	NR	NR
Parc et al. [41]	CJP	80	231.3 (89.8)	264	80	NR	NR (0–1.5)	5.9 (0.91)	NR	80/0
	SEA	87	217 (126.9)	260	87	NR	NR (0–0.6)	4.4 (0.96)	NR	87/0
Rasulov et al. [39]	CJP	22	185 (110, 280)	75 (30, 400)	11	NR	NR	NR	NR	NR
	SEA	30	230 (130, 340)	50 (30, 700)	8	NR	NR	NR	NR	NR
Okkabaz et al. [38]	CJP	29	213.1 (44.5)	200 (50, 1300)	29	8.5 (4.4)	8	(5, 6)	22/7	NR
	SEA	28	209.5 (50.1)	150 (50, 400)	28			(5, 6)	16/12	NR
Marković et al. [37]	CJP	40	NR	NR	40	NR	(2, 4)	7	40/0	0/40
	SEA	40	NR	NR	40	NR	(2, 4)	(3, 4)	40/0	0/40
Doeksen et al. [36]	CJP	55	NR	NR	18	3 (0.3, 5.8)	NR	(4, 6)	NR	17/34
	SEA	52	NR	NR	14	1.8 (0.3, 15.8)	NR	(3, 4)	NR	19/30
Akira et al. [35]	CJP	19	NR	NR	6	NR	4 (2.5, 6)	7 (5, 10)	NR	NR
	SEA	17	NR	NR	15	NR	4 (3, 6)	4.5 (3, 6)	NR	NR
Jiang et al. [34]	CJP	24	260.4 (22)	355.4 (61.5)	9	NR	5 (2, 7)	5 (0)	NR	24/0
	SEA	24	238.1 (12.8)	346.3 (45.9)	7	NR	5 (2, 7)	5 (0)	NR	24/0
Machado et al. [33]	CJP	65	NR	NR	NR	NR	NR	NR	NR	NR
	SEA	66	NR	NR	NR	NR	NR	NR	NR	NR
Machado et al. [32]	CJP	50	186 (115, 300)	500 (160, 1500)	1	NR	4 (2, 6)	8	NR	50/0
	SEA	50	197 (139, 375)	500 (100, 2250)	0	NR	4 (2, 6)	(3, 4)	NR	50/0
Huber et al. [31]	CJP	29	167 (130, 190)	NR	23	NR	3.8 (2, 5)	6	NR	NR
	SEA	30	149 (115, 175)	NR	21	NR	4.2 (3, 5.5)	(3, 4)	NR	NR

Continuous variables are recorded as mean (SD) or median (range). *CJP* colonic J-pouch, *SEA* side-to-end anastomosis, *NR* not reported

### Efficacy outcomes

All the included studies provided data about bowel function except the study by Rasulov et al. [39]. Three studies [36, 37, 40] could not be evaluated in the analysis because the bowel function data were evaluated by the validated Colorectal Functional Outcome (COREFO) questionnaire’s summary score, the modified version of the Memorial Sloan-Kettering Cancer Center (MSKCC) questionnaire, and composite evacuation/incontinence scores. The original data were not available even though we tried to contact the corresponding authors.

#### Bowel function

**Fig. 2:**
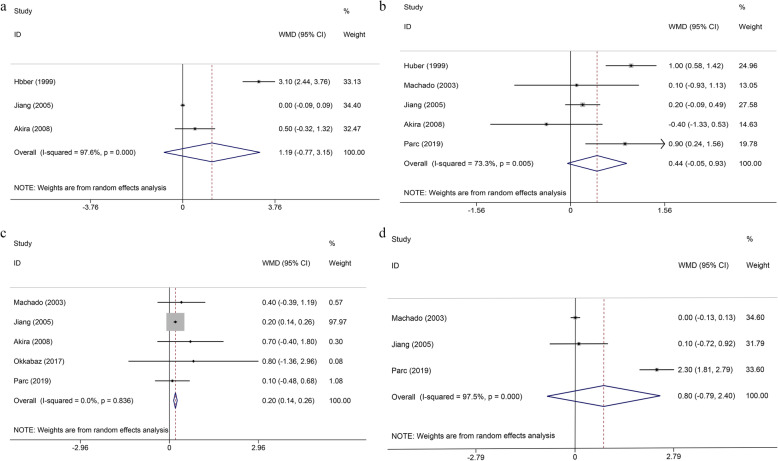
Forest plots of defecation frequency in different postoperative time. **a** 3 months, **b** 6 months, **c** 12 months, and **d** 24 months. WMD, weighted mean differences; CI, confidence intervals

In the pooled analysis of all 5 trials [31, 32, 34, 35, 41], the combination of defecation frequency in the CJP group was less than that in the SEA group at 12 months after SPR (WMD = 0.20; 95% CI, 0.14–0.26; *P* < 0.01), and there was no statistically significant between-study heterogeneity (*I*^2^ = 0%, *P* = 0.84). Random-effect analyses showed a trend towards less frequency at 3 months, 6 months, and 24 months after surgery. However, no significant differences were noted and heterogeneities were high (Fig. 2).

Of the included studies, four studies [31, 34, 35, 38] reported urgency, three studies [32, 34, 38] reported the rate of using pads, four studies [31, 34, 35, 38] reported the incomplete defecation, and three studies [31, 32, 34] reported the number of patients using enemas or other medications at 6 months after surgery. The available data pooled from two studies [31, 34] showed that SEA had benefits in terms of completeness of defecation 3 months after surgery (RR = 0.28; 95% CI, 0.09–0.86; *P* = 0.03). Other efficacy outcomes were not associated with the method of rectal reconstruction after SPR (Table 3).

**Table 3:** Bowel function of included trials

Bowel function (postoperative time/month)	No. of studies	SEA		CJP		Effect size		Heterogeneity	
		Events	Total	Events	Total	RR (95% CI)	*P*	*I*^2^	*P*
Urgency (early)^a^	4 [31, 34, 35, 38]	35	93	29	90	1.00 (0.74, 1.35)	1.00	0%	0.42
Urgency (intermediate)^b^	4 [31, 34, 35, 38]	24	93	16	90	1.05 (0.69, 1.61)	0.81	0%	0.40
Urgency (late)^c^	3 [34, 35, 38]	18	59	17	59	0.85 (0.59, 1.24)	0.41	0%	0.73
Pad using (intermediate)^b^	3 [32, 34, 38]	37	155	29	148	0.74 (0.42, 1.31)	0.30	0%	0.99
Pad using (late)^c^	3 [32, 34, 38]	29	150	29	146	1.39 (0.47, 4.14)	0.55	0%	0.52
Incomplete defection (3m)	2 [31, 34]	18	54	27	53	0.28 (0.09, 0.86)	0.03*	25%	0.25
Incomplete defection (6m)	3 [31, 32, 34]	36	98	34	97	1.15 (0.36, 3.65)	0.81	57%	0.10
Incomplete defection (12m)	2 [31, 34]	28	67	25	68	1.63 (0.62, 2.64)	0.55	0%	0.42
Enema (6m)	3 [31, 32, 34]	4	98	4	97	0.88 (0.19, 3.98)	0.87	0%	0.40
Medication (6m)	3 [31, 32, 34]	32	98	34	97	1.18 (0.42, 3.32)	0.75	41%	0.19

^a^Early, 3–4 months after surgery

^b^Intermediate, 6–8 months after surgery

^c^Late, 12 months after surgery

*P < 0.05

#### Anorectal manometry

**Fig. 3:**
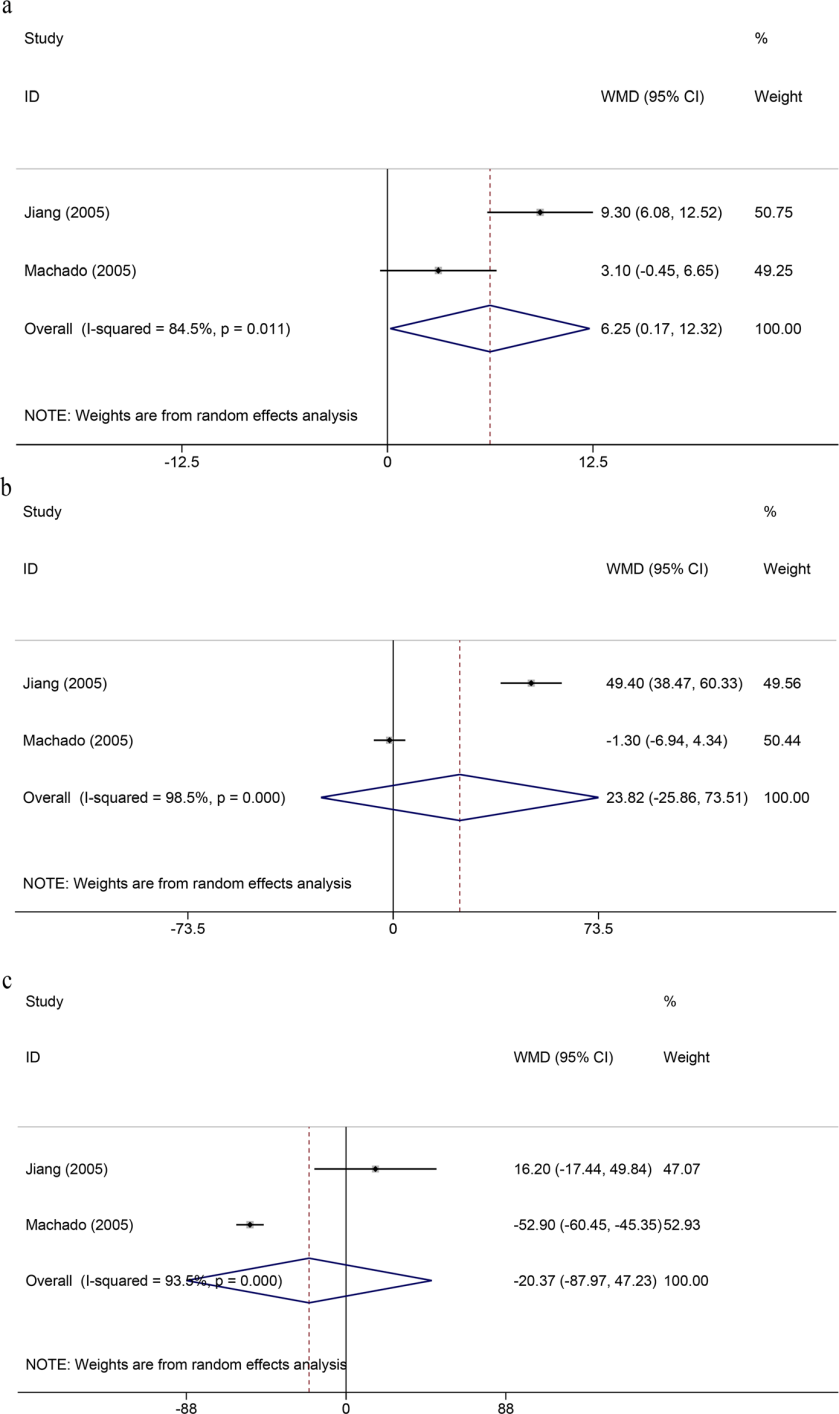
Forest plots of anorectal manometry. **a** Anorectal resting pressure. **b** Squeeze pressure. **c** Maximal tolerable volume. WMD, weighted mean differences; CI, confidence intervals

Four studies [31, 33–35] reported anorectal manometry data. However, meta-analysis could not be performed using all the studies. Huber et al. [31] and Akira et al. [35] reported their results with bar graphs, and the original data were not available. Pooled data from the other two studies [33, 34] that reported SEA were associated with high anorectal resting pressure (WMD = 6.25; 95% CI, 0.17–12.32; *P* = 0.04) but the heterogeneity was high (*I*^2^ = 84.5%, *P* = 0.01). We failed to find the source of heterogeneity due to the limitation of the included studies. Squeeze pressure and maximal tolerable volume were not associated with the method of rectal reconstruction after SPR (WMD = 23.83; 95% CI − 25.86 to 73.51; *P* = 0.35; WMD = − 20.37; 95% CI − 87.97 to 47.23; *P* = 0.56), and there was high heterogeneity (*I*^2^ = 98.5%, *P* < 0.001; *I*^2^ = 93.5%, *P* < 0.001) (Fig. 3). The anorectal manometry details are available in Additional file 3.

### Safety outcomes

#### Surgical outcomes

**Fig. 4:**
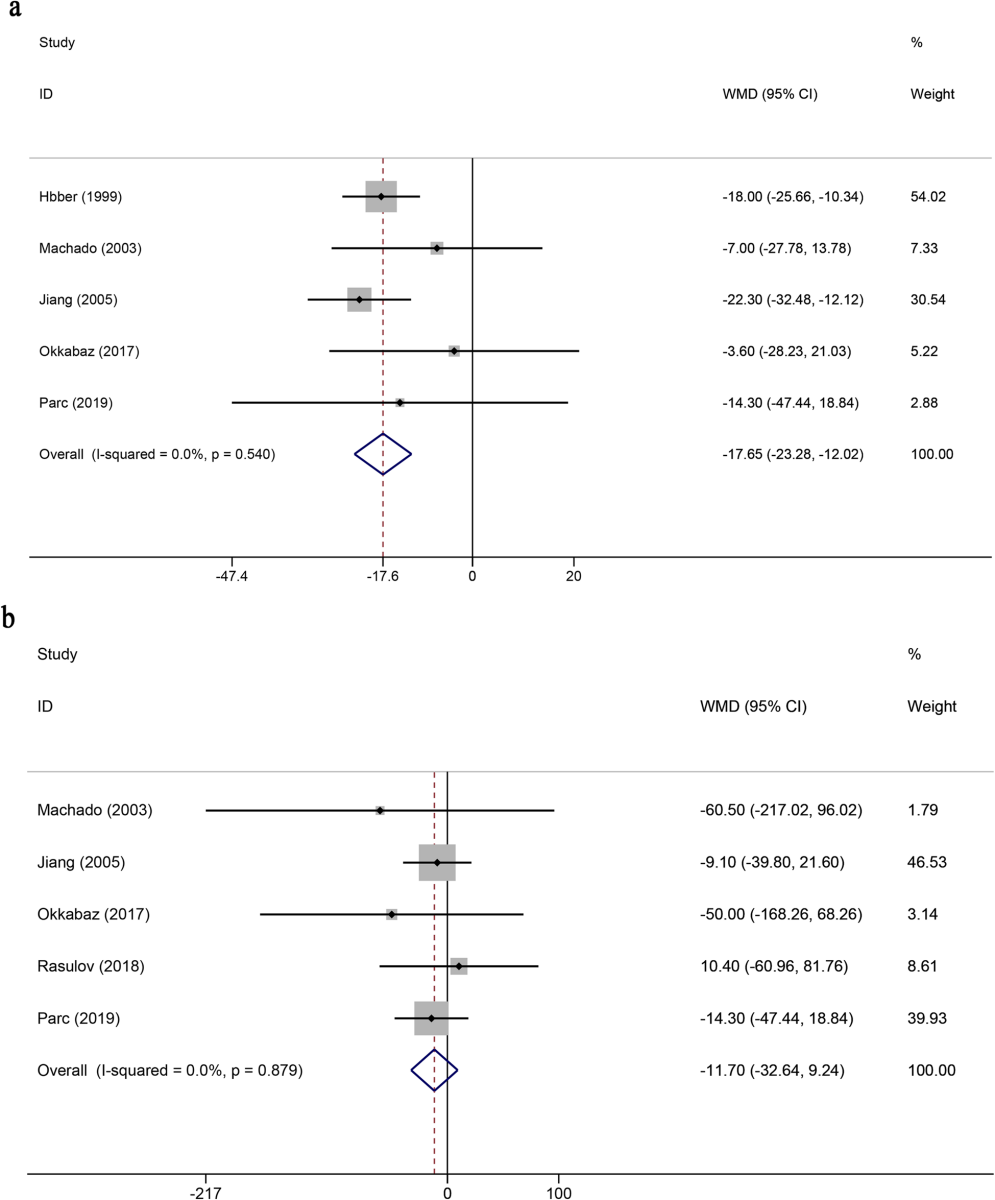
Forest plots of surgical outcomes. **a** Operating time. **b** Blood loss. RR, risk ratio; CI, confidence intervals

Pooled data from six studies [31, 32, 34, 38, 39, 41] reported operating times that showed no difference between SEA and CJP anastomosis (WMD = − 14.30; 95% CI, − 47.44 to 18.84; *P* = 0.40). When excluding one highly heterogeneous study [39] by the sensitivity analysis, SEA had a shorter operation time than CJP anastomosis (WMD = − 17.65; 95% CI, − 23.28 to − 12.02; *P* < 0.01) without significant heterogeneity (*I*^2^ = 0%, *P* = 0.54) (Fig. 4).

Blood loss was not affected by the two different anastomotic approaches (WMD = − 11.7; 95% CI, − 32.64 to 9.24; *P* = 0.27). Similarly, there was no heterogeneity between the included studies (*I*^2^ = 0%, *P* = 0.88) (Fig. 4).

**Fig. 5:**
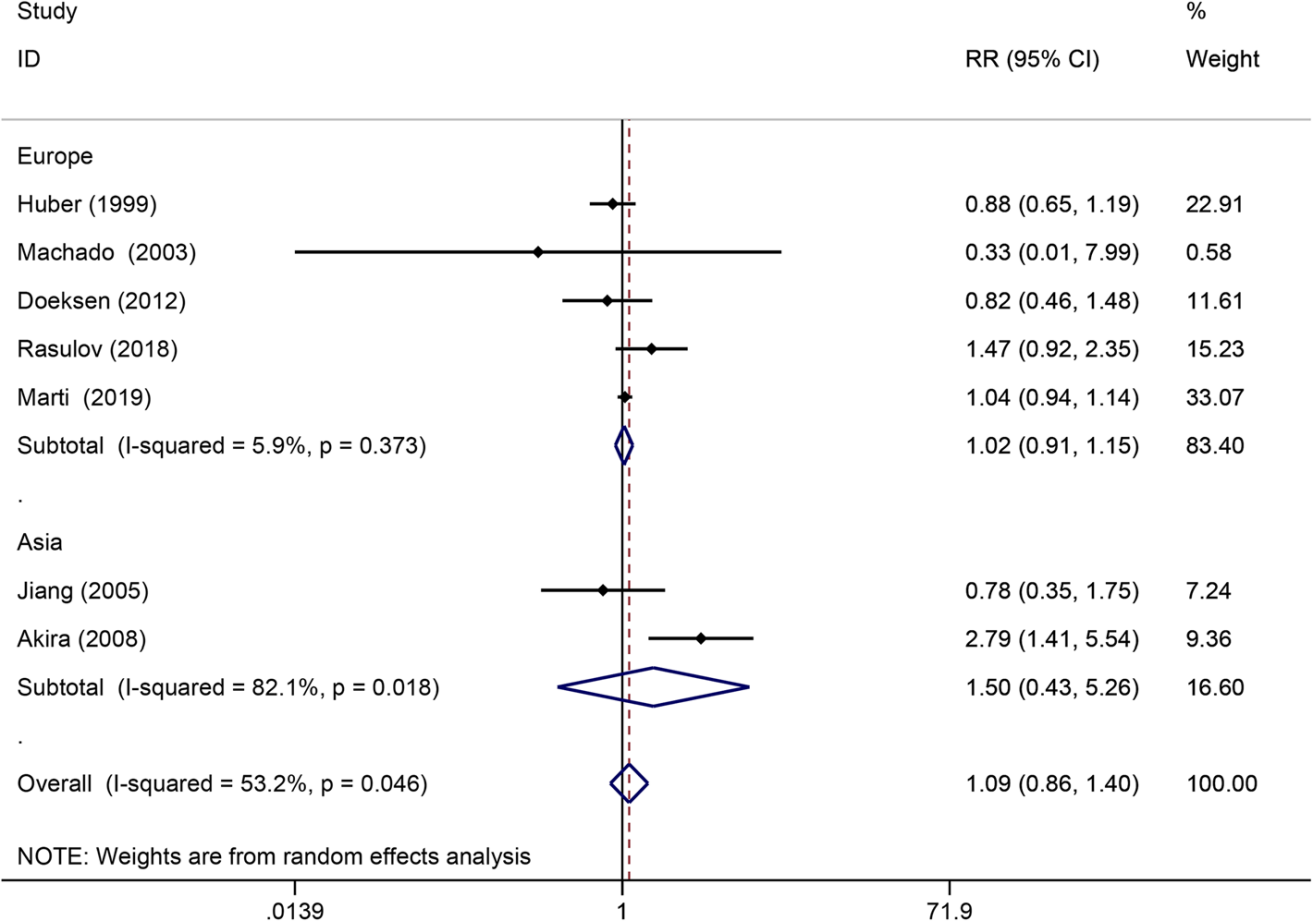
Forest plots of the protective stoma grouped by geographical location. RR, risk ratio; CI, confidence intervals

All 10 RCTs [31–41] included protective stoma. However, the meta-analysis was performed including only seven RCTs [31–36, 39, 40] because the other three RCTs [37, 38, 41] showed that all patients were treated with protective stoma. No significant differences were observed among the groups (RR = − 1.09; 95% CI, 0.86–1.40; *P* = 0.47). However, the heterogeneity was significant (*I*^2^ = 53.2%, *P* < 0.05). The subgroup analysis revealed that the use of protective stoma was still comparable between SEA and CJP anastomosis in Europe (RR = 1.02; 95% CI, 0.91–1.15; *P* = 0.69) and Asia (RR = 1.50; 95% CI, 0.43–5.26; *P* = 0.52) (Fig. 5). Sensitivity analysis and cumulative analysis revealed that the heterogeneity was caused by one study with the smallest sample size [35].

#### Clinical and oncological outcomes

No significant differences were observed between the groups in terms of anastomotic leakage (RR = 0.68; 95% CI, 0.41–1.13; *P* = 0.14), anastomotic stricture (RR = 0.97; 95% CI, 0.29–3.30; *P* = 0.96), pouch-related complications (RR = 0.50; 95% CI, 0.13–2.30; *P* = 0.32), pelvic sepsis (RR = 1.77; 95% CI, 0.87–3.60; *P* = 0.11), intestinal obstruction (RR = 1.10; 95% CI, 0.53–2.24; *P* = 0.83), wound infection (RR = 1.18; 95% CI, 0.39–3.62; *P* = 0.77), rectovaginal fistula (RR = 0.95; 95% CI, 0.24–3.79; *P* = 0.94), urinary complication (RR = 0.72; 95% CI 0.31–1.69; *P* = 0.53), cardiovascular complications (RR = 1.55; 95% CI, 0.31–7.76; *P* = 0.62), pneumonia (RR = 0.79; 95% CI, 0.16–3.85; *P* = 0.77), and postoperative hospital stay (WMD = 0.42; 95% CI, − 1.72 to 2.56; *P* = 0.70). Random model analysis revealed that oncological outcomes including in-hospital mortality, reoperation and relapse were not significantly different between the two rectal reconstructions with low heterogeneity (Table 4).

**Table 4:** Clinical and oncological outcomes of included trials

Clinical/oncological outcomes	No. of studies	SEA		CJP		Effect estimate		Heterogeneity	
		Events	Total	Events	Total	RR/WMD (95% CI)	*P*	*I*^2^	*P*
Anastomotic leakage	7 [31–36, 39, 40]	20	279	33	281	0.68 (0.41, 1.13)	0.14	0%	0.42
Anastomotic stricture	3 [32, 34, 41]	4	165	4	158	0.97 (0.29, 3.25)	0.96	0%	0.19
Pouch-related complications	4 [31, 34, 36, 41]	1	188	4	187	0.50 (0.13, 1.96)	0.32	0%	0.63
Pelvic sepsis	4 [31, 32, 36, 38]	17	151	10	158	1.77 (0.87, 3.59)	0.11	0%	0.84
Intestinal obstruction	4 [32, 36, 38, 41]	13	208	13	209	1.08 (0.53, 2.24)	0.83	0%	0.43
Wound infection	4 [32, 36, 38, 41]	6	208	5	209	1.18 (0.39, 3.62)	0.77	0%	0.84
Rectovaginal fistula	3 [31, 34, 41]	3	145	3	137	0.95 (0.24, 3.79)	0.94	0%	0.42
Urinary complications	3 [32, 34, 36]	8	121	12	128	0.72 (0.31, 1.69)	0.45	0%	0.53
Cardiovascular complications	2 [32, 36]	3	93	2	100	1.55 (0.31, 7.76)	0.59	0%	0.62
Pneumonia	2 [32, 36]	2	93	3	100	0.79 (0.16, 3.85)	0.77	0%	0.51
Mortality (in-hospital/< 30days)	3 [32, 36, 41]	5	189	3	185	1.53 (0.43, 5.45)	0.51	39%	0.20
Reoperations (except stoma reversal)	3 [32, 36, 38]	17	130	25	134	0.74 (0.36, 1.52)	0.41	24.7%	0.27
Relapse (< 2 year)	2 [36, 38]	4	80	4	84	1.05 (0.27, 4.03)	0.947	0%	0.99
Postoperative hospital stay (day)	4 [32, 36, 38, 39]	-	160	-	156	0.42 (− 1.72, 2.56)	0.70	30.5%	0.23

### Cumulative meta-analyses, sensitivity analyses, and subgroup analyses

Cumulative meta-analyses, sensitivity analyses, and subgroup analyses (according to the geographical location or sample size) were implemented to investigate the heterogeneity of operating time; protective stoma use; defecation frequency in the 3rd, 6th, and 24th month; incomplete defecation in the 3rd and 6th month; and anorectal manometry. Studies by Akira et al. [35] and Rasulov et al. [39] were the main drivers of heterogeneity in this review. Furthermore, sensitivity analysis revealed that the inclusion of the Asian study [34, 35] and fewer than 60 patients affected the heterogeneity or stability of the variables.

### Publication bias

We performed Harbord’s and Egger’s tests to assess the publication bias of the included studies. Funnel plots, Harbord’s test, and Egger’s tests demonstrated no evidence of publication bias (*P* > 0.05) (Additional file 4).

## Discussion

The present study showed that compared to CJP anastomosis, SEA had a shorter operating time and a higher anorectal resting pressure but a higher incidence of frequency in the 12th month after surgery. No significant difference was observed in the rate of protective stoma use, perioperative complications, postoperative hospital stay, mortality, reoperations, relapse, urgency, incomplete defecation, pad use, enema, or medication between the two surgical approaches.

The operating time in SEA was shorter than that in CJP anastomosis; the possible reasons were as follows. First, functional results in patients with either a short side limb (3 cm) or a long limb (6 cm) in SEA showed no significant difference [42]. Surgeons do not need to pursue a tension-free anastomosis at the expense of time-consuming colonic mobilization. Second, side-to-side anastomosis of the distal colon is unnecessary in SEA. However, this procedure may increase the operating time of CJP anastomosis. Third, operating time may increase in some patients with a narrow pelvis who underwent CJP anastomosis [15, 43]. In contrast, narrow operating space was not a limitation for SEA.

The rates of protective stoma use for the two anastomotic methods were not significantly different in our meta-analysis. After the subgroups were divided according to geographical location, we found that heterogeneity did not exist in the European studies. One Asian study [35] was excluded after cumulative analysis and sensitivity analysis were performed. In this study, 88.7% (15/17) of patients in the SEA group were treated with protective stoma compared to 31.6% (6/19) of patients in the CJP group. The small sample size may be related to the difference between groups. The pooled data of the remaining studies revealed that the rate of protective stoma use was independent of anastomotic methods. Impaired anorectal function was the main overall reason for a permanent stoma [44]. Okkabaz et al. [38] found that stoma closure could not be achieved in 28.1% of patients, with 11 (37.9%) in the CJP group and 5 (17.9%) in the SEA groups (*P* = 0.092). Although the difference was not statistically significant, more high-quality clinical trials are needed to determine whether CJP anastomosis is a risk factor for permanent stoma use.

The defecation frequency has not been previously analyzed quantitatively in published meta-analyses. Machado et al. [38], Jiang et al., and Okkabaz et al. [38] reported no significant difference in stool frequency between the CJP and SEA groups. However, in this study, SEA was associated with more bowel movements with low heterogeneity at the 12th month after SPR. The mechanism by which CJP anastomosis reduces defecation frequency has not yet been thoroughly studied. In 1999, Marco et al. [45] conducted an experimental study in the pig model. The difference of median neorectal compliance between small CJP anastomosis and SEA was significant (17.8 ml/mmHg vs 11.8 ml/mmHg, *P* < 0.001). Increased rectal compliance may contribute to fecal retention by decreasing the frequency [46].

Postoperative incomplete evacuation was more frequent in the J-pouch group than in the SEA group [31, 34], which was supported by a previous animal experiment [45]. The median times required for complete evacuation were 14 min and 4 min for CJP anastomosis and SEA, respectively. In our meta-analysis, SEA had no advantages over CJP in terms of evacuation. However, the few included studies and high heterogeneity made the evidence limited.

Suggested explanations for LARS include impaired neorectal capacity, decreased compliance, and the loss of rectal sensation [33, 47, 48], which could be evaluated by anorectal manometry. Anal resting and squeeze pressures help determine the presence of internal and external anal sphincter dysfunction [49]. Abnormally low resting pressure and no relaxation during rectal distension generally indicated that patients have severe weakness of the anal sphincter [47]. Our study showed that CJP anastomosis was associated with lower anorectal resting pressure and worse function of the anal sphincter. However, our evidence is insufficient because only two articles are included and the source of heterogeneity could not be analyzed.

In this meta-analysis, there was no heterogeneity for the majority of the safety and efficacy outcomes. We also tried to explain the potential sources of heterogeneity. Therefore, the pooled results were stable and conclusive.

There are several limitations to this study. First, it was not possible to obtain data for several key comparisons, such as the incidence of diarrhea, constipation, and incontinence. Other functional outcomes such as defecate at night, fragmentation, and differentiation between flatus and feces were reported in only one particular publication, thereby preventing a meta-analysis. Long-term outcomes such as overall survival and disease-free survival and sexual and bladder function, which are closely related to the quality of life of rectal cancer patients were not studied in this meta-analysis. Second, the analysis of anorectal manometry and incomplete defecation contained only two or three publications. Even though each trial had various strategies and different patient characteristics, the heterogeneity cannot be discussed further. Third, the current findings were based mainly on single-center small sample studies. Considering the limited quality of evidence for most outcomes, the use of SEA might be recommended as an alternative under some circumstances. Finally, we await long-term data from several ongoing studies to contribute to a more robust analysis of long-term quality of life.

## Conclusion

In conclusion, the current evidence suggests that SEA may be an effective strategy to expedite the surgical process without increasing the risks of surgery in SPR. Postoperative bowel functions of SEA were comparable to those of CJP anastomosis. However, close attention should be paid to the potential risk of frequent defecation. Because the present findings were based mainly on single-center studies, further powered studies are required to assess the implementation of this anastomotic technique.

## Supplementary Information

**Additional file 1:.** Registration of the protocol in PROSPERO.

**Additional file 2:.** Details of the literature search.

**Additional file 3:.** Details of anorectal manometry.

**Additional file 4:.** Publication bias assessment. a. Funnel plots of publication bias. b. Harbord test of publication bias. c. Egger test of publication bias.

## Data Availability

The datasets used and analyzed during the current study are available from the corresponding author on reasonable request.

## Abbreviations

SEA: Side-to-end anastomosis
SPR: Sphincter-preserving resection
CJP: Colonic J-pouch
RCTs: Randomized controlled trials
RR: Risk ratio
WMD: Weighted mean differences
CI: Confidence intervals
APRs: Abdominoperineal resections
LARS: Low anterior resection syndrome
SCA: Straight colorectal anastomosis
PROSPERO: International Prospective Register of Systematic Review
COREFO: Colorectal Functional Outcome
MSKCC: Memorial Sloan-Kettering Cancer Center
