# Supplementary material for: Safety and efficacy of side-to-end anastomosis versus colonic J-pouch anastomosis in sphincter-preserving resections: an updated meta-analysis of randomized controlled trials
Source: World J Surg Oncol. 2021 Apr 21;19:130. doi: 10.1186/s12957-021-02243-0 (PMC8061176; doi:10.1186/s12957-021-02243-0)
Supplement: Supplementary file 1 — Additional file 1:. Registration of the protocol in PROSPERO. [file 12957_2021_2243_MOESM1_ESM.pdf]

The efficiency and safety of side to end anastomosis for rectal reconstruction after low anterior resections: a meta-analysis of randomized controlled trials

Sen Hou, Quan Wang, Fan Liu, Yingjiang Ye

To enable PROSPERO to focus on COVID-19 registrations during the 2020 pandemic, this registration record was automatically published exactly as submitted. The PROSPERO team has not checked eligibility.

Citation

Sen Hou, Quan Wang, Fan Liu, Yingjiang Ye. The efficiency and safety of side to end anastomosis for rectal reconstruction after low anterior resections: a meta-analysis of randomized controlled trials. PROSPERO 2020 CRD42020206764 Available from:
https://www.crd.york.ac.uk/prospero/display_record.php?ID=CRD42020206764

Review question

It is reported 52%-76% of patients will suffer anorectal dysfunctions including fecal incontinence, urgency, frequency, constipation from low anterior resections (LAR) with total mesorectal excision (TME). The combination of abnormal clinical manifestations after LAR was referred as “low anterior resection syndrome (LARS)". Side to end anastomosis (SEA) is one of rectal reconstructions in low anterior resections. Some literatures have proved that side to end anastomosis can alleviate LARS. As the evidence of the evaluation of SEA was limited, we aimed to carry out a meta-analysis to investigate the safety and efficiency of SEA compared with colonic J-pouch anastomosis (CJP) and straight colorectal anastomosis (SCA).

Searches

PubMed, Cochrane, Web of Science, and Embase will be searched. Only literatures in English will be enrolled.

Types of study to be included

RCT.
Inclusion criteria: (1) studies included SEA group and at least a control group; (2) results including at least one outcome of interest.
Exclusion criteria: (1) less than 10 patients; (2) studies from the same institution or with overlapping patients;(3) not in English.

Condition or domain being studied

bowel reconstruction in rectal surgery

Participants/population

Inclusion criteria:(1) rectal cancer patients were clinically diagnosed and were performed sphincter-preserving surgery; (2) age?18

Exclusion criteria: (1) loss to follow-up (less than 3 months)

Intervention(s), exposure(s)

Side to end anastomosis in low anterior resections.

Comparator(s)/control

Colonic J pouch anastomosis and straight colonic anastomosis

Main outcome(s)

Main outcomes: (1)results of anorectal function after SEA (including the times of bowel movements during daytime and night, fecal incontinence, defection incomplete, use of pad, enema, and son on);(2) surgical complications of SEA.

* Measures of effect

relative risks, odds ratios, WMD

Additional outcome(s)

the pressure and volume of "new-rectum" after SEA

* Measures of effect

relative risks, odds ratios, WMD

Data extraction (selection and coding)

Two investigators independently will review the titles and abstracts of all citations identified by the literature

search. Eligible articles are reviewed for a duplicate in an independent manner by the two investigators.

Disagreement in data extraction is resolved by consensus.

Two authors independently extracted the following data: study demographics and characteristics, including: (1) first author, (2) publication year, (3) study design, (4) demographic of patients, (5) country, (6) multicenter or not, (7) date of inclusion, (8) tumor hight (cm) (9) tumor stage,(10) neoadjauvant and adjauvant therapies; safety outcomes, including: (1) operation time (min), (2) blood loss (ml), (3) length of hospital stay (days), (4) time to flatus (days), (5) time to liquid (days), (6) time to soft diet (days), and (7) number of patients with postoperative complications; and efficiency outcomes, including: (1) number of bowel movements, (2) defection frequency or not, (3) defection incomplete or not,(4) use pad or not,(5) use medicine or not, (6) urgency or not and so on

Risk of bias (quality) assessment

We use a funnel plot to detect publication bias concerning this meta-analysis, with the symmetry of the

funnel plot used to determine whether publication bias occurred. Furthermore, a formal statistical

assessment of the funnel plot asymmetry is performed with Begg's regression asymmetry test.

Strategy for data synthesis

All statistical analyses will be conducted using the statistical software Stata (version 16). The mean

difference, standard deviation, and standard error of the surgical time, blood loss, bowel movements are used for the meta-analysis. The number of adverse events happened and not happened will be analyzed to evaluate anotectal function and surgical complications.

Analysis of subgroups or subsets

neoadjuvant therapy, tumor stage, sex, and preoperative anorectal function.

Contact details for further information

Yingjiang Ye
yeyingjiang@pkuph.edu.cn

Organisational affiliation of the review

Peking university people's hospital, China
https://www.pkuph.cn/

Review team members and their organisational affiliations

Dr Sen Hou. Department of Gastrointestinal Surgery, Peking University People`s Hospital, Beijing 100044, PR China
Dr Quan Wang. Laboratory of Surgical Oncology, Peking University People`s Hospital, Beijing 100044, PR China Beijing Key Laboratory of Colorectal Cancer Diagnosis and Treatment Research, Peking University People`s Hospital, Beijing 100044, PR China
Dr Fan Liu. Laboratory of Surgical Oncology, Department of Gastrointestinal Surgery, Peking University

People's Hospital
Professor Yingjiang Ye. Department of Gastrointestinal Surgery, Peking University People`s Hospital, Beijing 100044, PR China

Type and method of review
Intervention, Meta-analysis, Systematic review

Anticipated or actual start date
29 August 2020

Anticipated completion date
01 November 2020

Funding sources/sponsors
National Natural and Science Foundation of China (NNSFC)

Grant number(s)

State the funder, grant or award number and the date of award

81871962

Conflicts of interest

Language
English

Country
China

Stage of review
Review Ongoing

Subject index terms status
Subject indexing assigned by CRD

Subject index terms
MeSH headings have not been applied to this record

Date of registration in PROSPERO
29 September 2020

Date of first submission
29 August 2020

Stage of review at time of this submission
The review has not started

Stage	Started	Completed
Preliminary searches	No	No
Piloting of the study selection process	No	No
Formal screening of search results against eligibility criteria	No	No
Data extraction	No	No
Risk of bias (quality) assessment	No	No
Data analysis	No	No

The record owner confirms that the information they have supplied for this submission is accurate and complete and they understand that deliberate provision of inaccurate information or omission of data may be construed as scientific misconduct.

The record owner confirms that they will update the status of the review when it is completed and will add publication details in due course.

Versions

29 September 2020

PROSPERO
This information has been provided by the named contact for this review. CRD has accepted this information in good faith and registered the review in PROSPERO. The registrant confirms that the information supplied for this submission is accurate and complete. CRD bears no responsibility or liability for the content of this registration record, any associated files or external websites.
