# Supplementary material for: Safety and efficacy of side-to-end anastomosis versus colonic J-pouch anastomosis in sphincter-preserving resections: an updated meta-analysis of randomized controlled trials
Source: World J Surg Oncol. 2021 Apr 21;19:130. doi: 10.1186/s12957-021-02243-0 (PMC8061176; doi:10.1186/s12957-021-02243-0)
Supplement: Supplementary file 2 — Additional file 2:. Details of the literature search. [file 12957_2021_2243_MOESM2_ESM.pdf]

SEARCH STRATEGIES

1.Pubmed:

((((((((((((((((Rectal Neoplasms[Title/Abstract]) OR (Neoplasm, Rectal[Title/Abstract])) OR (Rectal Neoplasm[Title/Abstract])) OR (Rectum Neoplasms[Title/Abstract])) OR (Neoplasm, Rectum[Title/Abstract])) OR (Rectum Neoplasm[Title/Abstract])) OR (Rectal Tumors[Title/Abstract])) OR (Rectal Tumor[Title/Abstract])) OR (Tumor, Rectal[Title/Abstract])) OR (Neoplasms, Rectal[Title/Abstract])) OR (Cancer of Rectum[Title/Abstract])) OR (Rectum Cancers[Title/Abstract])) OR (Rectal Cancer[Title/Abstract])) OR (Cancer, Rectal[Title/Abstract])) OR (Rectal Cancers[Title/Abstract])) OR (Rectum Cancer[Title/Abstract])) OR (Cancer, Rectum[Title/Abstract])) OR (Cancer of the Rectum[Title/Abstract]) And (side-to-end[Title/Abstract]) OR (side to end[Title/Abstract]) OR (end to side[Title/Abstract])OR(end-to-side[Title/Abstract] OR(Baker[Title/Abstract])

2. COCHRANE

#1 (side-to-end):ti,ab,kw OR (side to end):ti,ab,kw OR (end to side):ti,ab,kw OR (end-to-side):ti,ab,kw (Word variations have been searched)

#2 MeSH descriptor: [Rectal Neoplasms] this term only

#3 #1 AND #2 in Cochrane Reviews, Trials

3. Web of science

#1 [TS=(Rectal Neoplasm OR Neoplasm, Rectal OR Rectal Neoplasm OR Rectum Neoplasms OR Neoplasm, Rectum OR Rectum Neoplasm OR Rectal Tumors OR Rectal Tumor OR Tumor, Rectal OR Neoplasms, Rectal OR Cancer of Rectum OR Rectum Cancers OR Rectal Cancer OR Cancer, Rectal OR Rectal Cancers OR Rectum Cancer OR Cancer, Rectum OR Cancer of the Rectum)]

#2 [TS= (side-to-end OR side to end OR end to side OR end-to-side OR Baker)

#1 and #2

4.Embase

#3. #1AND #2

#1. 'Rectal Neoplasms' OR 'Neoplasm, Rectal' OR 'Rectal Neoplasm' OR 'Rectum Neoplasms' OR 'Neoplasm, Rectum' OR 'Rectum Neoplasm' OR 'Rectal Tumors' OR 'Rectal Tumor' OR 'Tumor, Rectal' OR 'Neoplasms, Rectal' OR 'Cancer of Rectum' OR 'Rectum Cancers' OR 'Rectal Cancer' OR 'Cancer, Rectal' OR 'Rectal Cancers' OR 'Rectum Cancer' OR 'Cancer, Rectum' OR 'Cancer of the Rectum'

#2. 'side to end' OR 'end to side' OR 'baker'
