# Supplementary material for: Safety and efficacy of side-to-end anastomosis versus colonic J-pouch anastomosis in sphincter-preserving resections: an updated meta-analysis of randomized controlled trials
Source: World J Surg Oncol. 2021 Apr 21;19:130. doi: 10.1186/s12957-021-02243-0 (PMC8061176; doi:10.1186/s12957-021-02243-0)
Supplement: Supplementary file 3 — Additional file 3:. Details of anorectal manometry. [file 12957_2021_2243_MOESM3_ESM.docx]

Additional file 3: Table S1: Maximal resting pressure (mmHg) of included studies

| Studies | Groups | Number | Pre-operation | Post-operation (3m) | Post-operation (6m) | Post-operation (12m) | Post-operation (24m) |
| --- | --- | --- | --- | --- | --- | --- | --- |
| Akira et al. 2008 [35] | CJP | 19 | 71.4 | 59 | 68.3 | 69.7 | NR |
|  | SEA | 17 | 81.6 | 76 | 80.7 | 69 | NR |
| Jiang et al. 2005 [34] | CJP | 24 | 61.3 (4.1) | 53.1 (4.1) | 55.7 (3.8) | 54.5 (4.0) | 54.0 (5.0) |
|  | SEA | 24 | 66.8 (4.4) | 59.0 (3.6) | 62.2 (4.0) | 66.8 (4.1) | 63.3 (4.7) |
| Machado et al. 2005 [33] | CJP | 36 | NR | NR | NR | NR | 42 |
|  | SEA | 35 | NR | NR | NR | NR | 47 |
| Huber et al. 1999 [31] | CJP | 29 | 45 | 36 | 39 | NR | NR |
|  | SEA | 30 | 46 | 38 | 40 | NR | NR |

Continuous variables are recorded as mean (SD) or median (range). Abbreviations: CJP, colonic J-pouch; SEA, side-to-end anastomosis; NR, not reported

Additional file 3: Table S2:: Maximal contraction pressure (mmHg) of included studies

| Studies | Groups | Number | Pre-operation | Post-operation (3m) | Post-operation (6m) | Post-operation (12m) | Post-operation (24m) |
| --- | --- | --- | --- | --- | --- | --- | --- |
| Akira et al. 2008 [35] | CJP | 19 | 240.7 | 204.2 | 217.9 | 222.1 | NR |
|  | SEA | 17 | 271.4 | 252.1 | 286.4 | 237.1 | NR |
| Jiang et al. 2005 [34] | CJP | 24 | 184.4 (15.1) | 170.6 (15.7) | 168.1 (11.0) | 178.0 (17.1) | 148.5 (13.6) |
|  | SEA | 24 | 224.4 (20.3) | 205.9 (16.5) | 219.6 (15.6) | 225.8 (21.8) | 207.1 (21.3) |
| Machado et al. 2005 [33] | CJP | 36 | NR | NR | NR | NR | 106 |
|  | SEA | 35 | NR | NR | NR | NR | 106 |
| Huber et al. 1999 [31] | CJP | 29 | 102 | 82 | 90 | NR | NR |
|  | SEA | 30 | 100 | 86 | 92 | NR | NR |

Continuous variables are recorded as mean (SD) or median (range). Abbreviations: CJP, colonic J-pouch; SEA, side-to-end anastomosis; NR, not reported
