# Supplementary figures and images for: Safety and efficacy of side-to-end anastomosis versus colonic J-pouch anastomosis in sphincter-preserving resections: an updated meta-analysis of randomized controlled trials
Source: World J Surg Oncol. 2021 Apr 21;19:130. doi: 10.1186/s12957-021-02243-0 (PMC8061176; doi:10.1186/s12957-021-02243-0)

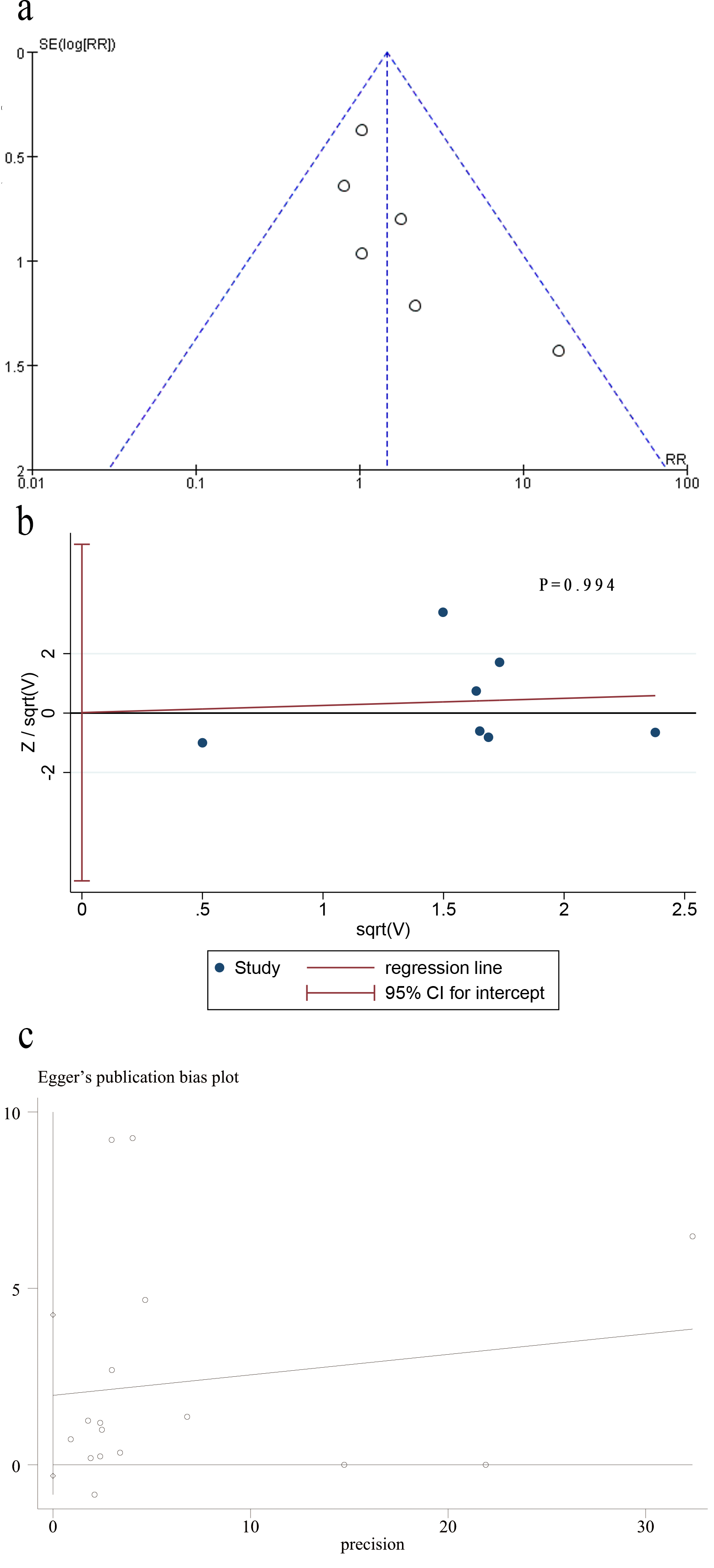

Supplement: Supplementary file 4 — Additional file 4:. Publication bias assessment. a. Funnel plots of publication bias. b. Harbord test of publication bias. c. Egger test of publication bias. [file 12957_2021_2243_MOESM4_ESM.tif]
